# Disruption of Asparagine Synthetase Is Associated to Increased Biomass in *Lotus japonicus*

**DOI:** 10.1111/pbi.70637

**Published:** 2026-03-24

**Authors:** Sara Rosa‐Téllez, Margarita García‐Calderón, David Barbosa Medeiros, Antonio J. Márquez, Alisdair R. Fernie, Marco Betti

**Affiliations:** ^1^ Departamento de Bioquímica Vegetal y Biología Molecular, Facultad de Química Universidad de Sevilla Sevilla Spain; ^2^ Institut de Biotecnologia i Biomedicina (BIOTECMED) Universitat de València Burjassot Spain; ^3^ Max Planck Institute for Molecular Plant Physiology Potsdam Germany

**Keywords:** asparagine synthetase, *Lotus japonicus*, metabolomics, nitrogen metabolism, rhizobial symbiosis

## Abstract

Asparagine (Asn) constitutes the major form of nitrogen translocated within *Lotus japonicus* plants. In this work we use knock‐out (KO) LORE1 mutants‐deficient in the asparagine synthetase gene (*LjASN1)*, which is the most highly expressed *ASN* gene in plants grown under non‐symbiotic (NS) conditions, but much less expressed under symbiotic (S) conditions. The analysis of two different *Ljasn1* homozygous mutant lines grown under NS or S conditions indicated that a much higher biomass was produced in *Ljasn1* mutants grown under NS conditions compared to the WT (wild‐type), whereas little difference with the WT was observed in mutant plants under S conditions. Metabolomic analysis revealed that *Ljasn1* mutant plants are quite distinct to WT plants when grown under NS conditions, but not under S conditions. Asn levels were considerably reduced in *Ljasn1* mutant plants compared to the WT when plants were grown under NS but not under S conditions. A general decrease in amino acids and an increase in carbon compounds, such as sugars and oxo‐acids, was detected in NS roots and shoots, respectively, which may explain the growth phenotypes observed. RNAseq analysis showed changes related to oxidative metabolism under NS conditions, and C/N metabolism under S conditions. The data indicate that the *LjASN1* deficiency produces important changes in the C/N balance and metabolite allocation of *L. japonicus* plants resulting in higher biomass content and lower Asn levels, two interesting traits for biotechnological crops engineering.

## 1 Introduction

Asparagine (Asn) is a key amino acid in plant metabolism, thanks to its high C/N ratio, and is used, among other things, for nitrogen transport and storage. In several plants, particularly temperate legumes, Asn is the major amino acid found in the xylem for root‐to‐leaves nitrogen transport, as well as in the phloem from the leaves to developing seeds (Lea et al. 2007) For example, in the model legume *Lotus japonicus*, Asn constitutes more than 80% of the nitrogen transported from root to shoot under N‐sufficient conditions (Waterhouse et al. 1996).

In addition to its function as an intra‐plant N‐transporting carrier from source to sinks, it has been reported that Asn can be a key signalling molecule that is used by the host plant for regulating the N‐flow between the nodule and sink organs in legumes (Sulieman and Tran 2013). Interestingly, it has also been shown that Asn is a key regulator in the N‐feedback loop modulating N_2_ fixation in other legume species (Sulieman et al. 2010). Other results also point out the relevance of Asn in soybean plants and possible agronomic applications of the manipulation of Asn metabolism (Antunes et al. 2008; Kambhampati et al. 2017 and references therein). Asn pool is reduced in legume nodules when symbiotic nitrogen fixation is partially arrested by N‐satiety conditions (Udvardi and Poole 2013), making this molecule a candidate as the primary sensor of the N status of the plant.

Asn metabolism has also received recent attention since accumulation of free (non‐protein) Asn in different organs of crop species is key for the formation of the highly toxic acrylamide when plant‐based products are cooked at high temperatures (Raffan et al. 2021). Breeding for low‐Asn crops is thus a highly promising area for biotechnological crops engineering. However, different agronomical traits other than Asn content must be taken into consideration when looking for varieties with lower Asn content through natural or induced variations (Oddy et al. 2022). Loss‐of‐function approaches aimed at causing a deficiency in the functional expression of genes encoding for asparagine synthetase (ASN), the main enzyme responsible for the biosynthesis of this amino acid, can lead to reduced Asn levels but are associated with non‐desirable phenotypes from an agronomic point of view including biomass reduction, reduced grain yield and grain protein content (Gaufichon et al. 2013; Luo et al. 2019; Lee et al. 2020).

Different works in our laboratory have analysed different aspects of Asn metabolism in *L. japonicus* (Credali et al. 2011, 2013) and have more recently revealed that there is a differential expression of genes involved in the metabolism of this amino acid in *L. japonicus* plants (García‐Calderón et al. 2017). *L. japonicus* has three genes encoding for *LjASN* (García‐Calderón et al. 2017). Of them, *LjASN1* is the most expressed in leaves, *LjASN2* shows a root and nodule specific pattern of expression, whereas *LjASN3* is expressed at low levels in all tissues (García‐Calderón et al. 2017). The expression of *LjASN1* is also light‐induced, suggesting a role for this gene in N‐transport in plants assimilating NO_3_^−^, since nitrate assimilation is maximal under light conditions (García‐Calderón et al. 2017).

In this work, we have carried out a loss‐of‐function approach to functionally characterise the *LjASN1* gene. The results obtained revealed a surprising increase in biomass in the mutant lines, but only when plants were grown under non‐symbiotic (NS) conditions. Several other molecular parameters such as Asn content, metabolic profiles and differential gene expression were also affected mainly under NS conditions. The results reveal a crucial role of this gene in C/N partitioning and have biotechnological interest given the high biomass and low Asn content of the knock‐out (KO) mutant lines.

## 2 Results

### 2.1 Isolation of LORE1‐Insertion Mutants in the *LjASN1* Gene

**FIGURE 1:**
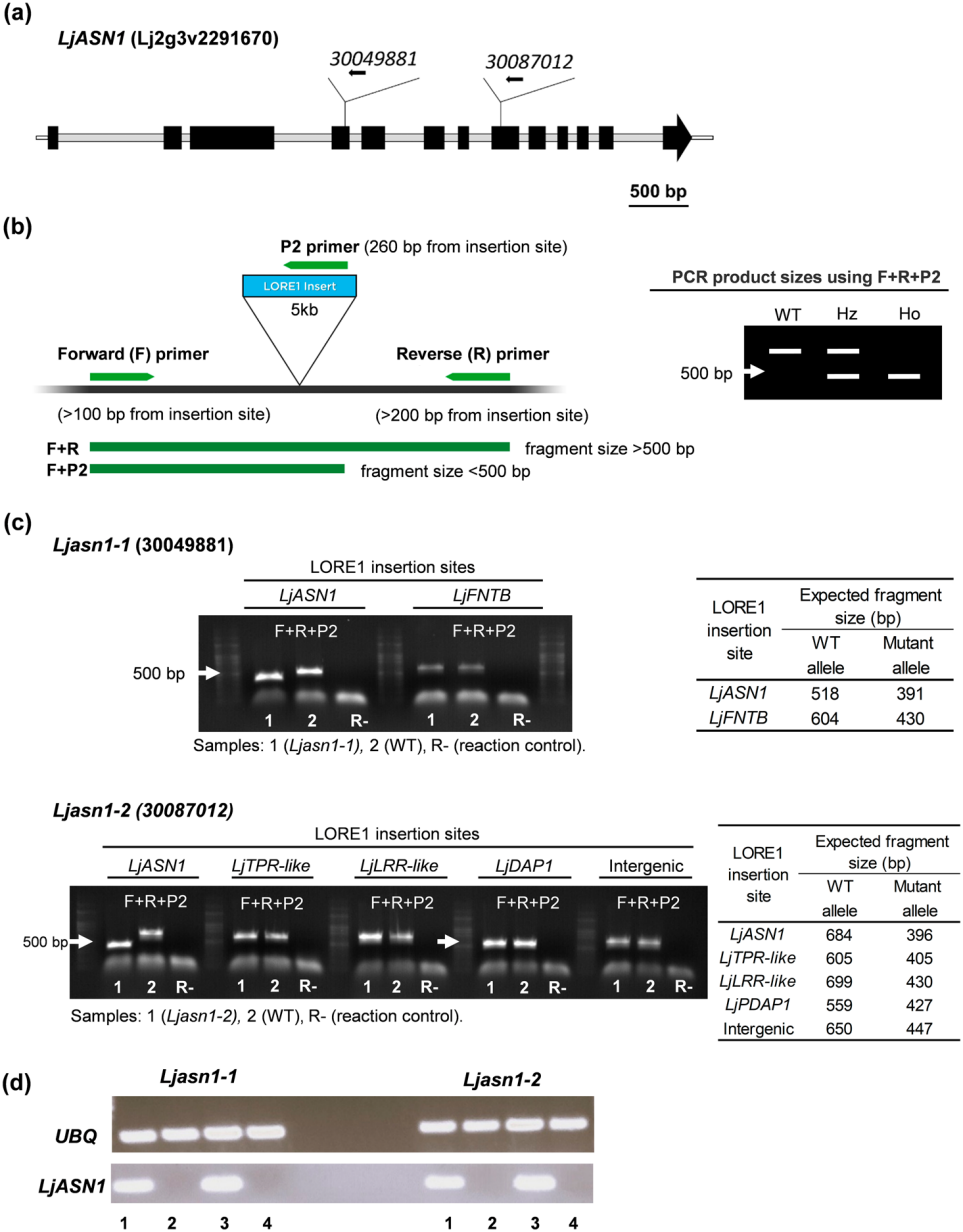
Genetic characterisation of the *Ljasn1* mutants. (a) Gene structure of *LjASN1* gene indicating the position of LORE1 transposable element insertion in the different *Ljasn1* mutant lines used in this work. The arrow indicates the orientation of the gene (from N terminal to C terminal). (b) Scheme of the strategy used for the genotyping of the LORE1 insertions. (c) Genotyping for the identification of the LORE1 insertions using a mix of the three primers (F, R and P2), demonstrating that the mutants are homozygous for the *LjASN* mutation and WT for the off‐target insertions. (d) Expression of the *LjASN1* gene in the two mutant lines. PCR reactions were carried out on cDNAs from WT and mutant plants using oligos specific for the *LjASN1* or the ubiquitin (UBQ) genes. Samples are: 1—WT shoots; 2—mutant shoots; 3—WT nodulated roots and 4—mutant nodulated roots.

To investigate the role of the *LjASN1* gene in *L. japonicus* , two independent mutant lines carrying insertions of the endogenous LORE1 retrotransposon were obtained from the *L. japonicus* LORE1 mutant collection (Fukai et al. 2012; Malolepszy et al. 2016). The two lines carried insertions in different positions of the Lj2g3v2291670 gene (or LotjaGi1g1v011820 in the Gifu 1.3 *L. japonicus* genome nomenclature) that encodes for *LjASN1*. Mutant line 30 049 881 (subsequently referred to as *Ljasn1‐1*) bears the insertion in the fourth exon of the gene while line 30 087 012 (subsequently referred to as *Ljasn1‐2*) bears the insertion in the eighth exon of *LjASN1* (Figure 1a). The seeds obtained from the LORE1 mutant collection are generated from self‐pollinated plants that carried insertions in the *LjASN* genes of interest and off‐target insertions (one genic insertion in the case of *Ljasn1‐1* and three genic insertions, plus one in the intergenic space, for *Ljasn1‐2*). To obtain clean mutants, genotyping was carried out to select homozygous mutants for the *LjASN1* mutation and WT for the off‐target insertions for both *Ljasn* lines according to the standard procedure described by Mun et al. (2017) (Figure 1b,c). The two independent *Ljasn1* homozygous lines were then self‐pollinated to obtain seeds and double‐checked for the homozygous insertion in the *LjASN1* gene and for the lack of insertions in the other positions before each experiment. RT‐PCR analysis was carried out to determine the expression levels of *LjASN1* in the mutants compared to WT plants. No *LjASN1* mRNA was detected in shoot or nodulated roots in both mutant lines (Figure 1d), so *Ljasn1‐1* and *Ljasn1‐2* can be considered KO mutants.

### 2.2 *Ljasn1* Mutants Show Increased Biomass Under NS Conditions

**FIGURE 2:**
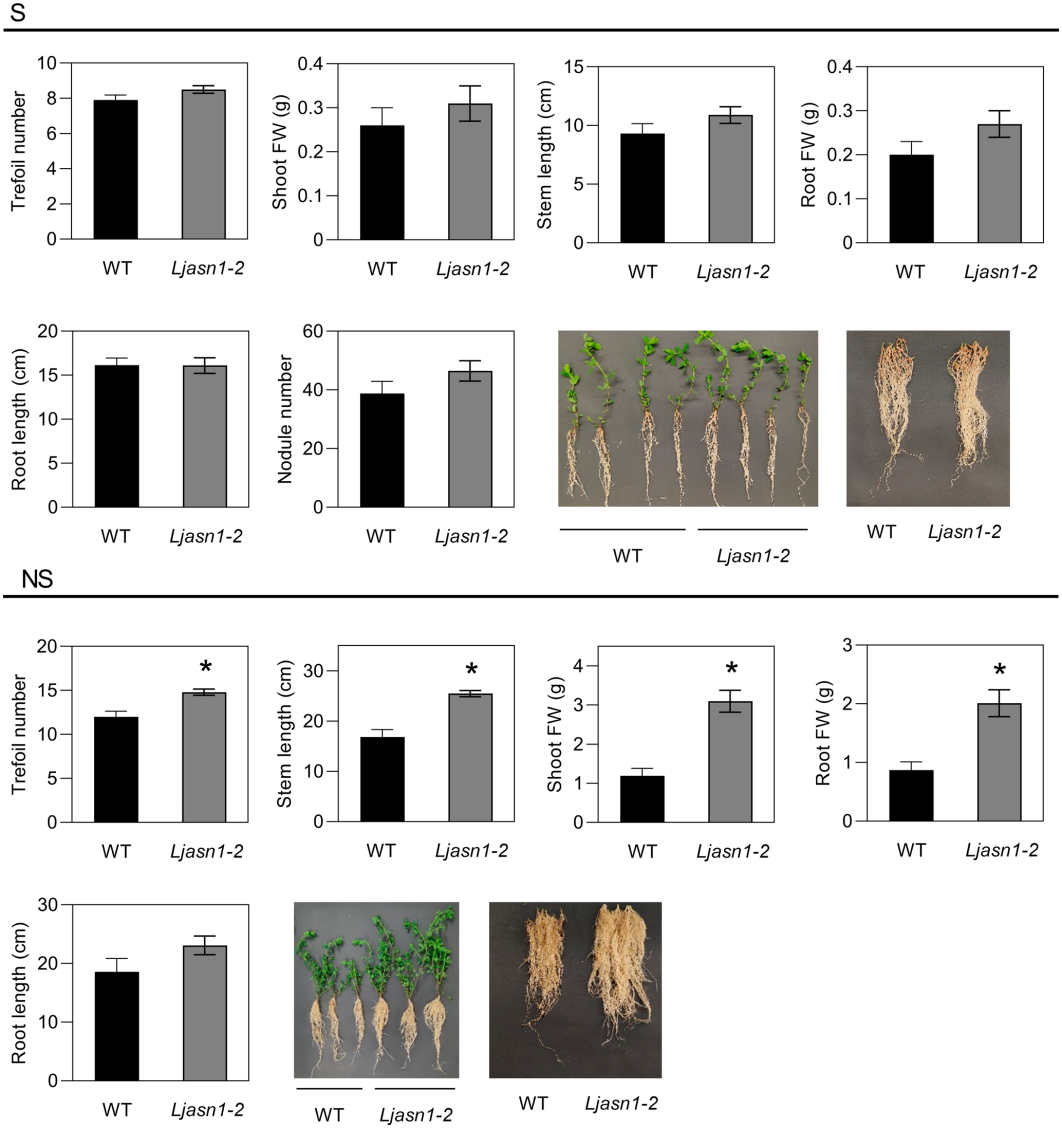
Growth parameters of WT and *Ljasn1‐2* mutant line under (a) symbiotic (S) conditions (b) non‐symbiotic (NS) conditions. Measurements were carried out after 7 weeks of growth and are the average ± SE of 10 independent plants. Pictures: Representative WT and *Ljasn1‐2* mutant plants and pool of roots from ten different plants for each genotype. Significant differences between WT and *Ljasn1‐2* mutant are denoted by an asterisk.

Different growth parameters for the WT and mutant lines were analysed for plants growing under symbiotic conditions (S), inoculated with *Mesorhizobium loti* , or NS conditions, in which an external nitrogen source was provided with the growth medium. Under S conditions, both mutant lines exhibited growth parameters like the WT plants (Figure 2, Figure S1). Nodulation efficiency was also like the WT in both mutant lines, as indicated by nodule biomass and acetylene reduction activity (Figure S2). However, under NS conditions both mutant lines showed much higher root and shoot biomass compared to the WT (Figure 2, Figure S1). Increased shoot biomass under NS conditions of the *Ljasn1* mutant lines was reflected in a higher trefoil number as well as increased total shoot biomass (2–3 times the WT one) and shoot length. The root system also showed a striking increase in biomass because of the *LjASN1* mutation. A total root weight of more than two times the WT was detected together with an increased root length (Figure 2, Figure S1). Since both mutant lines showed similar phenotypes, we selected the *Ljasn1‐2* mutant line for further molecular analysis.

### 2.3 Metabolomic Analysis Reveals Major Changes in N and C Metabolism Under NS Conditions

**FIGURE 3:**
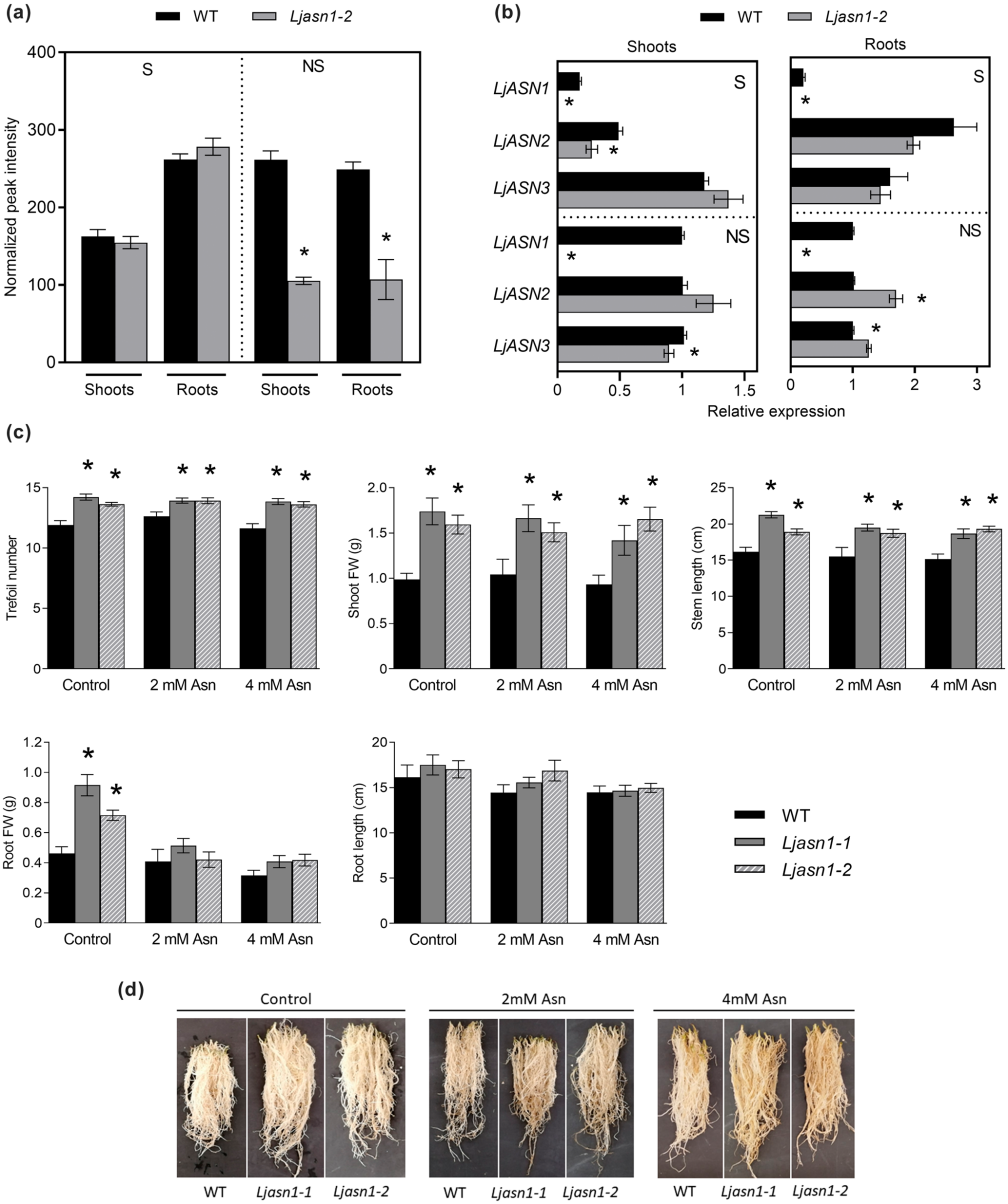
Asparagine (Asn) levels, gene expression analysis and growth parameters with external Asn feeding in WT and *Ljasn1* mutant plants. (a) Asn levels in shoots and roots of WT and *Ljasn1‐2* mutant plants grown under symbiotic (S) or non‐symbiotc (NS) conditions. Data represent the mean ± SE of 12 different biological replicates for the WT and 6 independent biological replicates for *Ljasn1‐2*. (b) RT‐PCR analysis of *LjASN* genes expression levels under S and NS conditions in shoots and roots of WT and *Ljasn1‐2* mutant plants. Data represent the mean ± SE of 3 different biological replicates. (c) Growth parameters of WT, *Ljasn1‐1* and *Ljasn1‐2* mutant lines under NS conditions in the absence (control) or presence of either 2 mM or 4 mM L‐Asn in the watering medium. Data are the average ± SE of 10 independent plants. Significant differences between WT and mutants at each [L‐Asn] are denoted with an asterisk. (d) A representative picture of WT and *Ljasn1* mutants pool of roots from ten different plants for each genotype at different [L‐Asn].

To gain insight into the metabolic consequences of the *LjASN1* mutation, a metabolomic analysis was carried out in shoots and roots of WT and mutant plants growing under S or NS conditions (see Tables S1 and S2 for shoots and roots, respectively). It could be clearly observed that Asn levels in *Ljasn1* mutants were less than half of the WT ones in shoots and roots of mutant plants growing under NS conditions, but no significant differences were observed when plants were grown under S conditions (Figure 3a). These results indicate that the presence of a functional *LjASN1* gene accounts for the majority of Asn levels detected under NS conditions, but very little, if any, under S conditions. Gene expression analysis was carried out in order to check if the other *LjASN* genes could compensate for the lack of *LjASN1*. Only a very slight induction of *LjASN2* and *LjASN3* in mutant roots under NS conditions was detected compared to the WT under the same conditions (Figure 3b), which was apparently not sufficient to compensate for the lack of *LjASN1* in terms of Asn levels (Figure 3a). On the other hand, these data indicate that LjASN2 and LjASN3 must be responsible for Asn biosynthesis under S conditions. To test if external Asn supply can restore the WT phenotype, plants were grown under NS conditions in the presence of 2 mM L‐Asn or 4 mM L‐Asn in the watering medium compared to 0 mM Asn of the control. In the presence of both Asn concentrations, the mutants showed WT‐like root growth, but the increase in mutant shoot biomass was still observed (Figure 3c,d, Figure S3).

**FIGURE 4:**
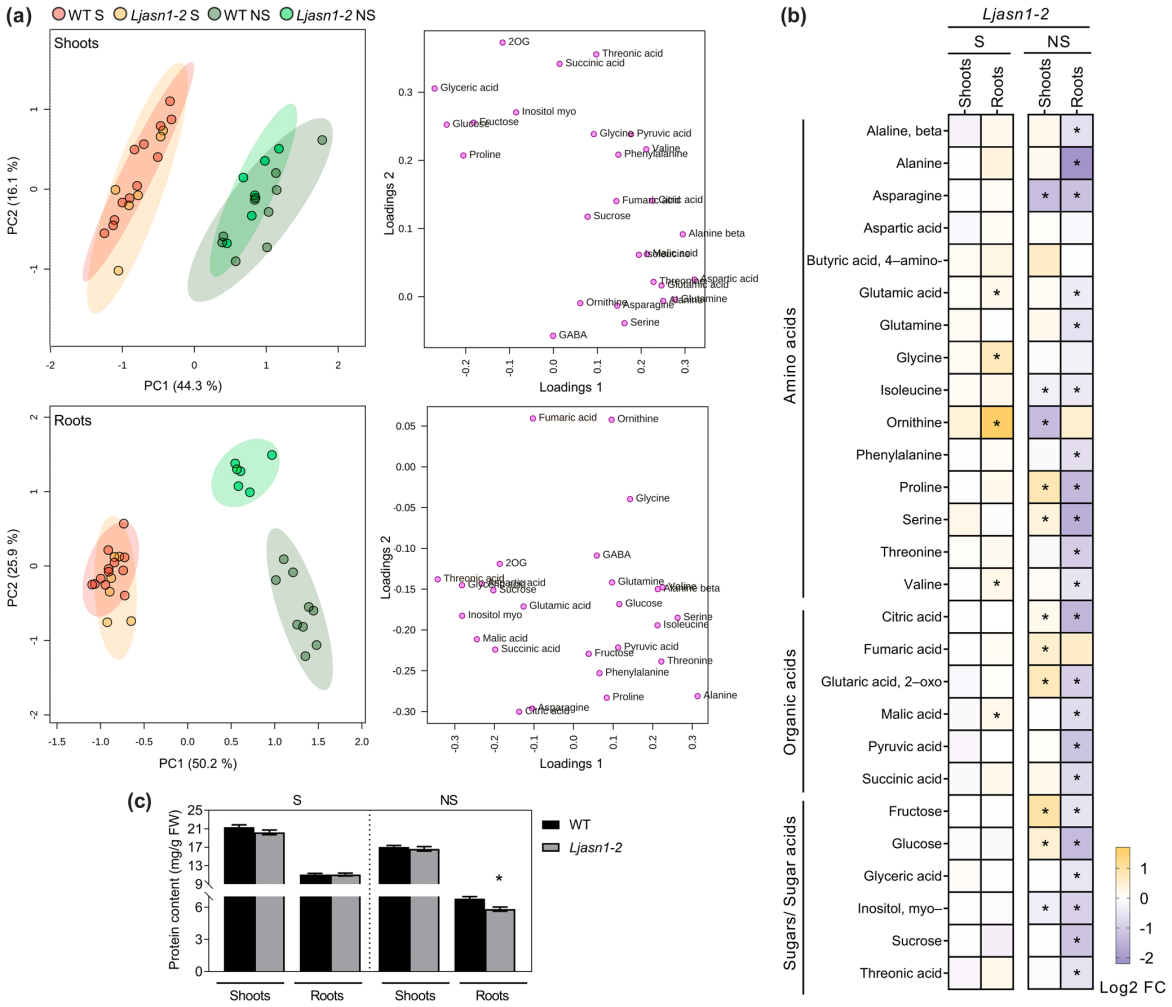
Metabolomic analysis of WT and *Ljasn1‐2* mutant plants. (a) Principal component analysis (left) and loading plots (right) of metabolomic profiling in shoots and roots of WT and *Ljasn1‐2* under S or NS conditions. (b) Heat map of metabolite changes in the Ljasn1‐2 mutant. Metabolite levels are represented in terms of log2 fold change compared to the WT. Metabolite showing significant changes in the mutant compared to the WT are indicated with an asterisk. (c) Total protein content in shoots and roots in WT (black bars) and *Ljasn1‐2* (grey bars) mutant plants under symbiotic (S) or non‐symbiotic (NS) conditions. Data represent the mean ± SE of 6 independent biological replicates of 10 plants each.

A Principal Component Analysis (PCA) was carried out to identify other metabolomic differences caused by the lack of a functional *LjASN1* gene. The analysis showed little differences between *Ljasn1‐2* and WT tissues under S conditions, but a clear separation between the metabolic profiles of WT and mutant roots under NS conditions (Figure 4a). Interestingly, other differences in the metabolome, but not related to *LjASN1* mutation, were explained by the growth conditions as can be depicted from the principal component 1 (PC1) in both shoots and roots, accounting for 44.3% and 50.2%, respectively (Figure 4a). In shoots, the profiles of WT and *Ljasn1‐2* were very similar under both growth conditions, but S and NS samples were well separated along the PC1 axis, meaning that the nutritional conditions rather than the genotype accounted for the metabolic variability in this tissue. In this regard, the most important changes observed were in sugars such as fructose, myo‐inositol, glyceric acid and the amino acids proline, aspartic acid, glutamic acid, glutamine and beta‐alanine, which showed an opposite trend depending on the nutritional condition (Figure 4a). In roots, WT and mutant metabolomic profiles clustered together under S conditions but were separated from samples from roots under NS conditions along the PC1 axis. However, WT and mutant root profiles were also separated along the PC2 axis (accounting for 25.9% of the variability), indicating major metabolic changes in roots as a consequence of the mutation exclusively under NS conditions (Figure 4a).

Figure 4b and Tables S1 and S2 further illustrate the changes detected in the relative levels of metabolites found to be of particular interest. Under S conditions, no significant differences in metabolite levels were detected between WT and the *Ljasn1‐2* mutant except for minor changes in the levels of some amino acids that were slightly increased and, more remarkably, ornithine that was increased about threefold in the mutant compared to the WT. However, a completely different metabolic profile could be found between WT and the *Ljasn1‐2* mutant in the case of NS plants. Interestingly, most amino acids, sugars and carbon metabolites were decreased in the mutant roots under NS conditions, in parallel with the lower levels of Asn detected in this genotype. However, in shoots, the *Ljasn1‐2* mutant showed an increase in several sugar metabolites such as glucose and fructose, as well as other carbon compounds such as 2‐oxoglutarate as compared to the WT plants (Figure 4b). Total protein levels were lower in mutant roots under NS conditions, but not S conditions (Figure 4c), consistent with the lower levels of amino acids detected in this tissue. In contrast, the total protein content was not different from the WT in mutant shoots under either nutritional condition (Figure 4c).

### 2.4 Transcriptomic Analysis Confirms Major Differences in *Ljasn1‐2* Mutants Under NS Conditions

**FIGURE 5:**
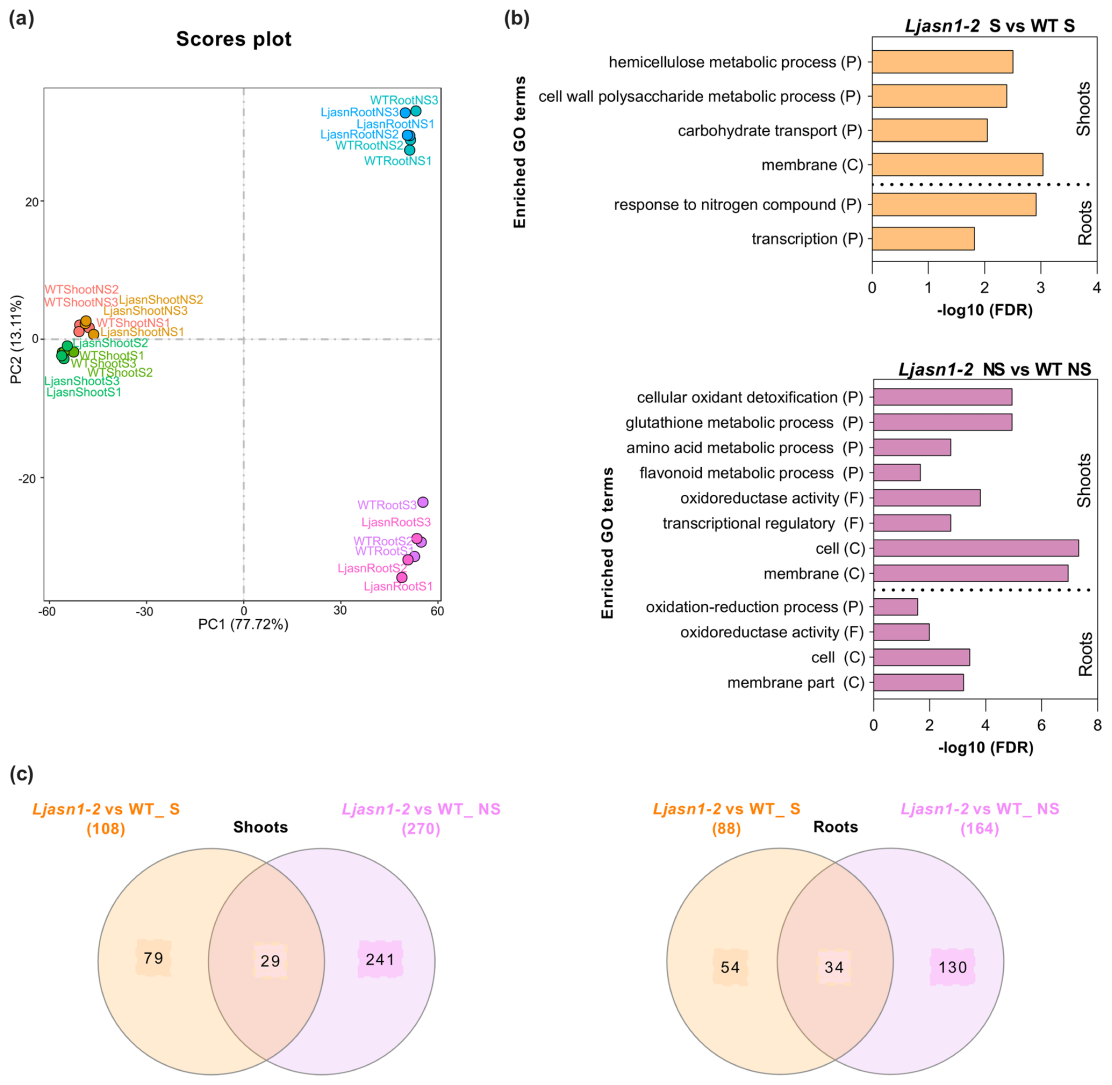
RNAseq analysis of WT and *Ljasn1‐2* mutant plants. (a) PCA analysis of transcriptome sequencing data according to the gene expression values (FPKM) in the different genotypes, tissues and nutritional conditions. (b) Enriched GO terms in mutant shoots and roots under symbiotc (S) or non‐symbiotic (NS) conditions. (c) Number of *Ljasn1* mutant DEGs (with respect to WT) which are also commonly or differentially expressed in S or NS growth conditions either in shoots or roots.

RNAseq analysis was carried out to investigate possible changes in gene expression occurring in the *Ljasn1‐2* mutant. Differential gene expression analysis was carried out by comparing WT and mutant plants using a corrected *p* value cut‐off of 0.05. The number of differentially expressed genes (DEGs) detected was much higher under NS than S conditions, as also generally happened for previously growth, metabolomic and protein parameters. PCA analysis of the gene expression values (FPKM) showed that all shoot samples clustered together whatever the genotype or growth condition (Figure 5a). The three replicates for the shoots of each genotype under both S and NS conditions clustered closely together; consequently, individual replicates occupy nearly the same space on the PCA plot (Figure 5a, left part of the graph). However, although root samples were separated on the PC2 axis based on the growth conditions, WT and *Ljasn1‐2* clustered together, indicating that the S or NS growth condition has more effect than the mutation on the global root gene expression.

Transcriptome sequencing identified 108 and 88 DEGs in shoots and roots, respectively, under S conditions, whereas 270 and 164 DEGs were found in shoots or roots under NS conditions (Tables S3 and S4). Under S conditions, the majority of DEGs were down‐regulated (26 up‐regulated and 82 down‐regulated in shoots, 15 up‐regulated and 73 down‐regulated in roots), whereas in NS conditions, shoot DEGs were mostly up‐regulated (158 up‐regulated and 112 down‐regulated DEGs) while root DEGs were mostly down‐regulated (56 up‐regulated and 108 down‐regulated). Gene Ontology (GO) Enrichment Analysis of DEG genes revealed that carbon and nitrogen metabolic pathways such as flavonoid and amino acid biosynthesis were significantly altered in *Ljasn1‐2* mutant plants, compared to the WT, mainly in NS conditions, in agreement with previous results. Under S conditions, most significantly enriched GO terms were found in shoots. Several categories of genes related to carbohydrate and cell wall metabolism were enriched (Figure 5b, Figure S4), which is in good agreement with the increase in sugars and C metabolites observed. In roots under S conditions, enriched GO terms were related to N metabolism and gene expression process, basically Zinc finger proteins and ubiquitination‐related enzymes (not shown). Under NS conditions, the most enriched GO terms were again found in the DEGs from mutant shoots and were mainly related to stress response, detoxification and antioxidant defences (Figure 5b, Figure S5). Among the genes in the enriched terms, several encoded for glutathione‐S‐transferase isoforms and enzymes for flavonoid decoration (mainly glycosyltransferases) were more expressed in the mutant. In roots, GO terms enrichment was less significant, but some terms related to oxidoreductase activity were also found. The high number of GO enriched pathways related to stress and oxidative defences was in part because some genes belong to more than one similar GO term. Several enriched ‘cellular component’ GO terms were found in the mutant shoot and root transcriptomes under NS conditions (Figure S5), which probably reflects increased cell growth and/or proliferation associated to the increased biomass observed in both tissues.

A further analysis was carried out to investigate if DEGs were common or exclusive for each nutritional condition (Figure 5c). In shoots, only 29 DEGs were common to S and NS conditions, whereas 34 DEGs were common in roots, representing 8.3% and 15.6% of the total DEGs in each tissue, respectively (Table S5). This indicates that most of the effect of the mutation at the transcriptional level is specific for either S or NS growth conditions, which is in good agreement with the different phenotypes observed for the mutant plants at the growth and metabolic levels. In agreement with the phenotypic and metabolomic data, most DEGs were differentially expressed exclusively under NS conditions (Figure 5c; 241 in shoots and 130 in roots). These DEGs exclusive to NS conditions were then subjected to GO enrichment analysis. In shoots, GO terms related to, among others, glucose metabolic process, sulphate reduction, cyanide metabolism and nucleoside transmembrane transport were upregulated. On the other hand, genes involved in pigment synthesis and the response to biotic and abiotic stress were downregulated (Table S5). In roots, genes related to glycine, serine and tetrahydrofolate metabolism were upregulated. Interestingly, an enrichment in genes involved in L‐arabinose and inositol metabolism was also found in mutant roots under NS conditions. These compounds are known to be present in plant root exudates to promote symbiosis. In contrast, genes involved in defence response, salicylic acid signalling pathway and transport of sulphate were downregulated (Table S5).

## 3 Discussion

The present paper provides different types of evidence to show that the disruption of one *LjASN* gene (*LjASN1*) in the model legume *L. japonicus* results in lower levels of free Asn, associated with changes in the metabolome and transcriptome of the plants, together with a considerable increase in shoot and root biomass.

While *Ljasn1* mutant roots exhibited lower Asn levels and a marginal increase in *LjASN2* and *LjASN3* expression under NS conditions (Figure 3a,b), the specific contribution of each isoform to the Asn pool remains to be fully elucidated. Future studies are needed in order to do that, taking into consideration that ASN polypeptide levels and enzyme activity are generally difficult to determine in plant extracts (Azevedo et al. 2006) including *L. japonicus* ones (Pérez‐Delgado et al. 2015).

Different plant species use different types of compounds for N‐translocation. Most plants, like Arabidopsis, use preferentially glutamine during light conditions, whereas they use Asn in dark (Coruzzi 2003). In contrast, tropical legumes such as soybean use preferentially ureides as N‐transport compounds, particularly under S conditions, but still Asn has also an intriguing role in tropical legume plants (Antunes et al. 2008; Kambhampati et al. 2017). However, some temperate legumes such as *L. japonicus* preferentially use Asn both in light and dark conditions (Waterhouse et al. 1996). *LjASN1* was shown to be a light‐inducible gene (García‐Calderón et al. 2017), suggesting a connection between this gene and the light‐dependent nitrate assimilation. In fact, *LjASN1* was the asparagine synthetase gene showing the highest level of expression in leaves and roots, both in light and dark conditions, particularly under ammonium nitrate NS growth conditions (García‐Calderón et al. 2017). Quite interestingly, although *Ljasn1* mutants grown under NS conditions had plenty of inorganic nitrogen available in the growth medium, their root metabolic profile, as well as the increased root growth phenotype may be indicative of internal N‐deficiency. Thus, it may be possible that the low Asn levels in the mutant roots can be a signal for N deficiency in *L. japonicus* .

Mutants in the *ASN1* gene from other plant species do not show a substantial increase in growth of both aerial and root systems (Gaufichon et al. 2017; Ohashi et al. 2015; Lee et al. 2020). Disruption of other *ASN* genes can lead to lower biomass paralleled by lower Asn levels (Gaufichon et al. 2013) or lower Asn levels but normal vegetative growth (Gaufichon et al. 2016; Raffan et al. 2021). In rice, mutation of *OsAS1*, mainly expressed in roots, was associated with a slight increase in shoot length and a decrease in root length at the seedling stage together with a drop of 80%–90% of free Asn under NH_4_^+^ nutrition (Ohashi et al. 2015). However, under N‐limiting conditions, *osasn1* mutants showed impaired growth and slightly lower biomass at the seedling stage (Lee et al. 2020). On the other hand, recent work has shown that the supply of exogenous Asn has a stimulating effect on Arabidopsis plant growth (Lardos et al. 2024). This effect is independent of the enantiomeric form of the amino acid, suggesting that the effect is due to the molecule per se, rather than its N content. Supplementing Asn or glutamine at low concentrations can also increase shoot length in *Phaseolus vulgaris* (Haorun et al. 2010), suggesting a positive correlation between Asn levels and plant growth rates. Supplementation with L‐Asn in the watering medium suppressed the increase in mutant root biomass, supporting the hypothesis that low Asn levels are responsible for this phenotype (Figure 3c,d). However, increased shoot biomass was still observed in mutants supplied with different concentrations of external Asn (Figure 3c, Figure S3). Further experiments with different Asn supplementation methods, concentrations and enantiomeric forms are needed to get a clearer picture of the relationship between Asn and plant growth in *L. japonicus* .

The phenotype of the *Ljasn1* KO mutant lines presented here indicates a role for LjASN1 mainly under NS conditions. Metabolomic data indicate a C/N imbalance in the mutants under NS conditions, as depicted from the lower amino acid levels and lower total protein in the NS roots, and higher sugar levels in the shoots. The phenotype of the NS roots can be a response to a situation detected by the plant as internal N‐deficiency, which must trigger increased root growth, possibly aimed to increase N foraging, whereas in the shoots the higher availability of sugars can be the determinant of the increase in biomass. Analysis of available datasets of root from WT plants under long‐term N starvation (≥ 10 days) is consistent with this hypothesis. A significant reduction in root amino acid levels, specifically glutamate, glutamine, aspartate and Asn was found under N starvation in Arabidopsis, tomato and sweet orange ( *Citrus sinensis* ) (Krapp et al. 2011; Renau‐Morata et al. 2021; Lai et al. 2023). Furthermore, mirroring our own findings, transcriptomes from N‐starved roots showed an overrepresentation of Gene Ontology (GO) terms related to stress and defence, ion transport, and amino acid metabolism (Krapp et al. 2011; Calabrese et al. 2017). Conversely, long‐term N‐starvation studies in WT plants have shown increased root levels of fructose, glucose and sucrose, reflecting carbon remobilisation to support root growth (Krapp et al. 2011; Renau‐Morata et al. 2021). In contrast, *Ljasn1* mutants exhibited decreased levels of these sugars. Furthermore, *Ljasn1* mutants did not display the changes in shoot‐to‐root biomass ratio typically observed under externally imposed N starvation. These results suggest that while the *LjASN1* mutation partially mimics the effects of N deficiency, the impact is not sufficient to trigger a shift in biomass allocation from shoots to roots.

PCA analysis of GC/MS data indicated that roots of WT and mutant plants under NS conditions were the most metabolically different samples (Figure 4a), suggesting that the phenotypes observed in the mutant shoots may be a consequence of the metabolic imbalance generated in roots. On the other hand, PCA analysis of RNAseq data showed again separate clustering of root samples, but the main variability was explained by the growth condition (NS or S) rather than the genotype (Figure 5a). This suggests that the transcriptional changes observed could be related to the symbiotic status in the S plants, or the ability to assimilate inorganic nitrogen in the NS plants. Nitrogen‐related transcriptomic changes observed in roots of the *Ljasn1* mutants could also be related to the fact that *L. japonicus* assimilates nitrate mainly in roots.

The lack of growth phenotype of the *Ljasn1* mutants under S conditions is probably related to the normal levels of Asn, in contrast to what is observed under NS conditions. However, some changes were observed mainly at the transcriptomic level also under S conditions, the most significant related to cell wall metabolism according to GO term analysis (Figure S4, Figure 5b). At the metabolomic level, the most differential compound was ornithine, which was increased more than three times in mutant roots under S conditions (Table S2). Ornithine is a central metabolite in plants and a precursor for proline and polyamines, constituting also a link between glutamate and arginine metabolism (Majumdar et al. 2016). Glutamate levels were also increased in the mutant roots under S conditions. Given the normal levels of Asn in the mutant under S conditions, and the lack of a direct link between Asn biosynthesis and ornithine, the increase in this amino acid, as well as glutamate, glycine and valine in the mutant S roots remains puzzling. The normal nodulation observed for the *Ljasn1* mutant, together with the lack of growth phenotype under S conditions, suggests that the *LjASN1* gene is not essential for symbiosis in *L. japonicus* , despite the changes observed. Although our data demonstrate that nodule functionality in the mutants remains uncompromised under S conditions (Figure S2), recent evidence suggests a specialised role for legume *ASN1* during nodule senescence. Specifically, *LjASN1* has been identified as a target of FUN transcription factors, which positively regulate nodule senescence (Lin et al. 2024), whereas soybean *ASN1* appears to facilitate nitrogen redistribution as symbiotic fixation declines (DelPercio et al. 2025). These recently discovered links may hint at a role for *LjASN1* under S conditions beyond the maintenance of the Asn pool, which may explain the differences at the omics level observed in the nodulated mutant. Further studies on the nodule senescence dynamics of *Ljasn1* mutants may help to define the precise contribution of *LjASN1* to this process.

**FIGURE 6:**
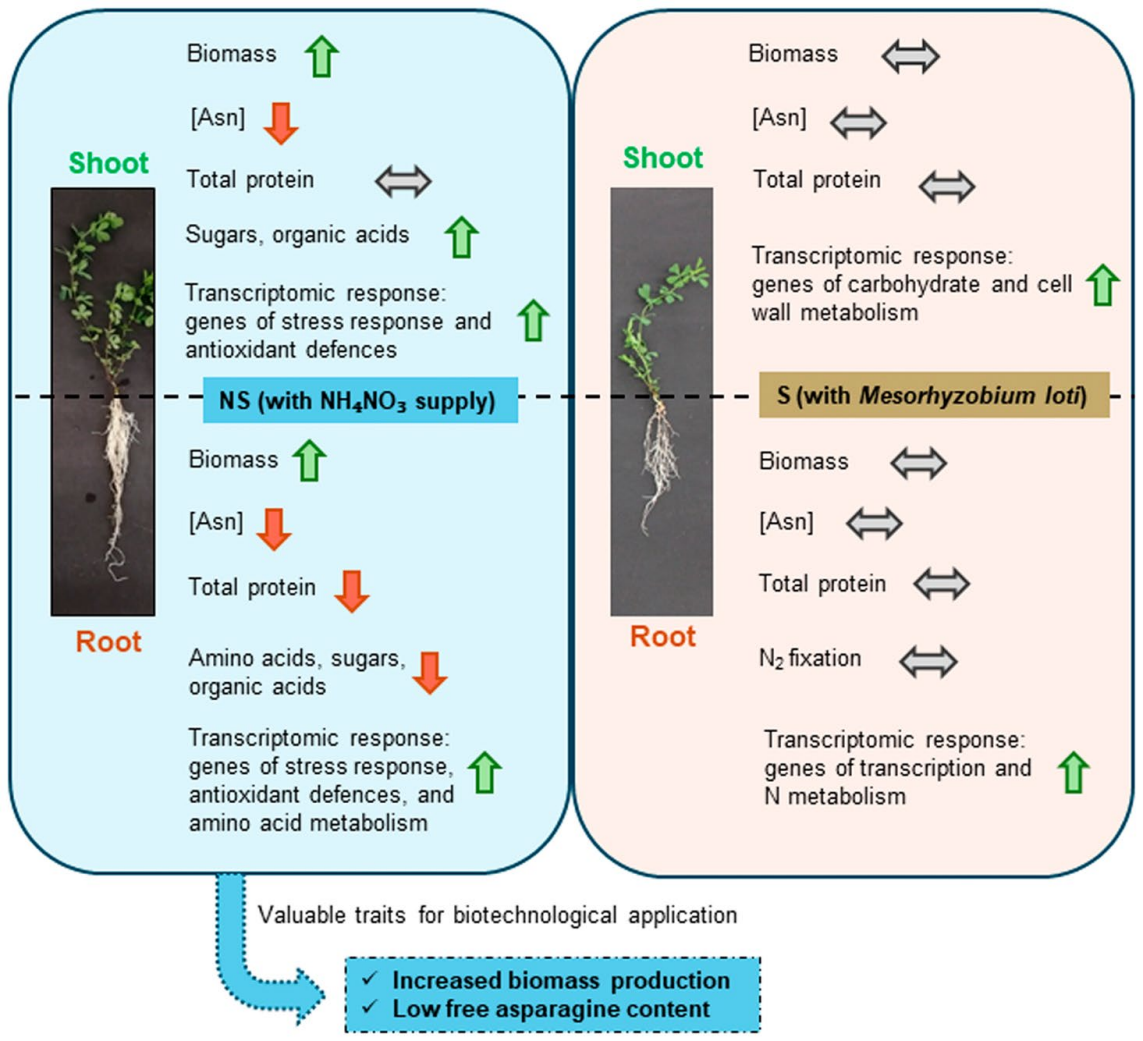
Schematic representation of the molecular and phenotypic consequences of *LjASN1* disruption under different nitrogen regimes: non‐symbiotic (NS) and symbiotic (S) conditions. Green upward arrows indicate an increase, red downward arrows denote a decrease, whereas grey horizontal lines indicate no significant change compared to the wild type under the same condition.

Manipulation of Asn metabolism by means of gene disruption or overexpression has been carried out with the aim of improving crop yield. Although overexpression of *ASN* genes can lead to positive agronomical traits such as increased grain yield and protein content, for example in lettuce (Giannino et al. 2008) and rice (Lee et al. 2020), this is also associated with increased Asn levels. In contrast, lower free Asn levels, such as those detected in *Ljasn1* mutants, are desirable for food safety. This is because free Asn and reducing sugars (or sucrose, if it is first hydrolysed) are the precursors for toxic acrylamide formation in plant‐based products (Halford et al. 2012). Therefore, a primary goal of current crop breeding is to lower free Asn content without compromising other agronomic traits (Ly et al. 2023; Raffan et al. 2023). In this study, while Asn levels were lower in the mutant shoots, glucose and fructose levels increased in *Ljasn1‐2* mutants under NS conditions (Table S1). However, since Asn is the major determinant of acrylamide formation potential (Halford et al. 2012; Oddy et al. 2022), the data indicate that the *Ljasn1* mutants possess valuable traits for biotechnological application: decreased Asn levels coupled with increased biomass production (Figure 6).

The *Ljasn1* root phenotype under NS conditions is of special interest since improved primary root length and total root biomass is a desirable trait to improve crop nitrogen capture from soils (Lynch et al. 2024). Although this phenotype was observed only under NS conditions, a potential for increased N uptake can be useful even for legumes growing in symbiosis. For example, in *L. japonicus* supplying low amounts of nitrate can promote nodulation (Barbulova et al. 2007), and the nodulation and growth of cultivated legumes such as soybean benefit, in field conditions, from inorganic nitrate uptake, providing that the nitrate concentration is not so high as to induce nodule senescence (Guillierme et al. 2025). To provide a comprehensive overview of the phenotype of the *Ljasn1* mutants, the key findings of this study are resumed into the schematic summary presented in Figure 6.

## 4 Conclusions

The data presented here demonstrate that *LjASN1* is not strictly required under S conditions in *L. japonicus* , but its impairment leads, under NS conditions, to increased biomass paralleled by low Asn levels; both very interesting phenotypes from an agronomic point of view. Although *L. japonicus* is a model species, the high levels of synteny that exist between the different legume genomes (Libault et al. 2009) imply that the advances obtained with this species can be used to improve the performance of cultivated legumes.

## 5 Experimental Procedures

### 5.1 Plant Material, Growth and Experimental Designs

The model legume *L. japonicus* (Regel) K. Larsen ecotype Gifu (B‐129‐S9) was used in all experiments. Seeds were originally obtained from Dr. Jens Stougaard (Aarhus University, Denmark) and self‐propagated at the University of Seville. Seeds from the progeny of self‐pollinated heterozygous plants carrying the LORE1 transposon inserting in the *LjASN1* gene were obtained from the LORE1 database (https://lotus.au.dk/) and homozygous mutant plants were selected by genotyping as described by Monje‐Rueda et al. (2023) using the primers listed in Table S6. In addition to the *LjASN1* gene, *Ljasn1‐1* seeds may carry insertions of the LORE1 retrotransposon in two additional genes, one exonic and one intronic, whereas *Ljasn1‐2* may have three ulterior insertions (one exonic and two intronics). To obtain ‘clean’ mutants, several plants from each line were genotyped by PCR to isolate homozygous plants for the *LjASN* insertion and lacking transposon insertions in the other genes.

Growth parameters were determined using five seedlings planted in each pot as a biological replicate and using vermiculite as a solid support and grown in a growth chamber under 16/8 h day/night, 20°C/18°C with a photosynthetic photon flux density of 200 μmol m^−2^ s^−1^ and a constant humidity of 70%. Plants growing under NS conditions were irrigated with ‘Hornum’ nutrient solution containing 5 mM NH_4_NO_3_ and 3 mM KNO_3_. Plants under S conditions were inoculated with the compatible symbiont *Mesorhizobium loti* and watered with nitrogen‐free ‘Hornum’ medium supplemented with 3 mM KCl (Handberg and Stougaard 1992). *Mesorhizobium loti* TONO JA76 (Kawaguchi et al. 2002) was grown in liquid YM medium at 28°C to an optical density of 1.0 at 600 nm, and then collected by centrifugation for 30 min at 2408 *g* and resuspended in 0.75% (w/v) NaCl. Once sown in the pots, the plants were inoculated by the addition of 200 μL of this bacterial suspension. Ten plants per genotype were analysed to determine the growth patterns under S and NS conditions.

For the Asn feeding experiment, WT and the two mutant lines were grown under NS conditions as described before, but with the addition of 2 mM or 4 mM L‐Asn in the watering Hornum medium, or with Hornum medium alone (control conditions with 0 mM Asn). Plants were watered every 3–4 days with the watering medium with or without the addition of L‐Asn, which was added from a filter‐sterilised 0.1 M stock solution. Ten plants per genotype and condition were analysed to determine the growth patterns.

### 5.2 Gene Expression Studies

For gene expression studies, plant tissues were flash‐frozen in liquid nitrogen, ground to a fine powder with mortar and pestle and stored at −80°C until use. Total RNA was isolated from *L. japonicus* leaves using a modified CTAB extraction method as described by Kistner and Matamoros (2005) and DNAse treated with the Turbo DNA‐free kit (Ambion). Plants were harvested at the same growth stage (26 days after planting for NS conditions and 37 days after planting for S conditions). RNA integrity and concentration were checked using an Agilent Bioanalyzer 2100 (Agilent Technologies), and a Nano‐Drop ND‐1000 (Nano‐Drop Technologies), respectively. RNA extractions were carried out with three independent biological replicates for each genotype/condition/tissue. Each replicate was composed of 10 plants grown in two different pots. The tissues were harvested separately after the 8‐h light period for analysis. For RNAseq analysis, library preparation and transcriptome sequencing were conducted by Novogene (Cambridge, UK). Differential gene expression analysis was performed using the DESeq2 R package. *p*‐values were adjusted using the Benjamini and Hochberg's approach and assigned as differentially expressed when *p*‐value < 0.05. Gene Ontology Enrichment Analysis was performed using the software AgriGO v2.0 (Tian et al. 2017) according to the hypergeometric test. RT‐PCR analysis was carried out according to Monje‐Rueda et al. (2023) using ubiquitin (*LjUbq4*), as reporter gene, that was selected from the most stably expressed genes in this plant species (Sanchez et al. 2008). A list of the primers used in this work is available as Table S6.

### 5.3 Metabolomic Analysis

Metabolomic analysis was carried out from the same plant tissues used for RNAseq studies, with the exception that biological replicates consisted of 10 individual plants from two different pots that were harvested together. Six independent biological replicates were analysed for the *Ljasn1* mutant. For the WT control, 10–12 replicates were used, comprising two distinct lines: the original Gifu (B‐129‐S9) and a syngenic WT line. The latter consisted of WT segregants lacking the *LjASN1* LORE1 insertion, derived from the original LORE1 seed population. Metabolite content in derivatized methanol extracts by GC–MS (Gas chromatography–mass spectrometry) using the protocol described by Lisec et al. (2006). Metabolites were identified in comparison to database entries of authentic standards (Kopka et al. 2005). Chromatograms and mass spectra were evaluated with the Chroma TOF 1.0 (LECO) and TagFinder 4.0 software (Luedemann et al. 2008).

### 5.4 Other Measurements

Proteins were extracted using an ethanolic extraction as described by Stitt et al. (1989). The total protein content was determined colorimetrically using the Bradford protein assay (Bio‐Rad) using bovine serum albumin (BSA) as the standard.

For determination of acetylene reduction activity, the root of each plant was separated from the aerial part and quickly placed into a 10 mL tube and sealed with a rubber stopper. Using a syringe, one‐tenth of the air volume was extracted from the container and replaced with the same volume of acetylene. After 1 h, three 1 mL samples were extracted from the atmosphere of each flask and analysed in a Shimadzu Q2010 gas chromatograph with flame ionisation detector and a Porapak T column of 1 m length. Acetylene reduction activity (ARA) is reported as nmol ethylene/hour × g nodule FW.

### 5.5 Statistical Data Analysis

Experimental data values are reported as means and standard error (SE), and *n* represents the number of independent samples. Significant differences between control and mutants were analysed by Student's *t*‐test using Microsoft Excel. Plots were generated in GraphPad 8 software.

### 5.6 Accession Numbers

*LjASN1* gene sequence can be downloaded from the Lotus Base information portal (http://lotus.au.dk) with the accession number LotjaGi1g1v011820, according to the last version of the *Lotus japonicus* v. Gifu genome. The two mutant lines used in this study (30049881, called *Ljasn1‐1* and 30087012, called *Ljasn1‐2*) can also be found at the Lotus Base portal.

## Author Contributions

A.J.M., M.G.‐C., M.B. and S.R.‐T. conceived and designed the experiments. S.R.‐T. and M.G.‐C. carried out the isolation and phenotypical characterisation of the mutants. D.B.M. and S.R.‐T. performed GC–MS experiments. S.R.‐T. performed the analysis of the GC–MS data. M.B., M.G.‐C. and S.R.‐T. performed RNAseq analysis. M.B. and A.J.M. drafted the manuscript. S.R.‐T. and M.G.‐C. produced the figures. All the authors contributed to the writing of the final version of the manuscript.

## Funding

This work was supported by grant PID2021‐122353OB‐I00 funded by MICIU/AEI/10.13039/501100011033 and FEDER, UE and RTI‐2018‐093571‐B100 from MICIU/AEI/10.13039/501100011033 and FEDER, UE. Open Access funding provided by the Universidad de Sevilla.

## Conflicts of Interest

The authors had complete control of the design of the study, the collection, analysis and interpretation of data, the writing of the manuscript and the decision to publish, without interference from the funders and partner organisations listed in the acknowledgements.

## Supporting information


**Figure S1:** Growth parameters of WT and *Ljasn1‐1* mutant line under (a) S conditions or (b) NS conditions.

**Figure S2:** Nodulation parameters of WT and mutant lines.

**Figure S3:** A representative picture of WT and mutant plants after feeding of different [L‐Asn] under NS conditions.

**Figure S4:** List of enriched GO terms among the shoot and root DEGs under S conditions.

**Figure S5:** List of enriched GO terms among the shoot and root DEGs under NS conditions.

**Table S1:** Metabolite relative content in the shoots of WT and *Ljasn1‐2* plants grown under NS or S conditions.

**Table S2:** Metabolite relative content in roots of WT and *Ljasn1‐2* plants grown under NS or S conditions.

**Table S3:** List of genes differentially expressed between *Ljasn1‐2* and WT under S conditions.

**Table S4:** List of genes differentially expressed between *Ljasn1‐2* and WT under NS conditions.

**Table S5:** DEGs classified according to the criteria used for the VENN diagram representation in Figure 5c, that is, if they are differentially expressed in the mutant under both nutritional **conditions** or exclusively under S or NS conditions.

**Table S6:** List of the oligonucleotides used in this work.

## Data Availability

The RNAseq data that support the findings of this study are openly available in NCBI's Gene Expression Omnibus, reference number GSE283121 (https://www.ncbi.nlm.nih.gov/geo/query/acc.cgi?acc=GSE283121).
