# Supplementary figures and images for: Disruption of Asparagine Synthetase Is Associated to Increased Biomass in Lotus japonicus
Source: Plant Biotechnol J. 2026 Mar 24;24(7):4471–83. doi: 10.1111/pbi.70637 (PMC13278535; doi:10.1111/pbi.70637)

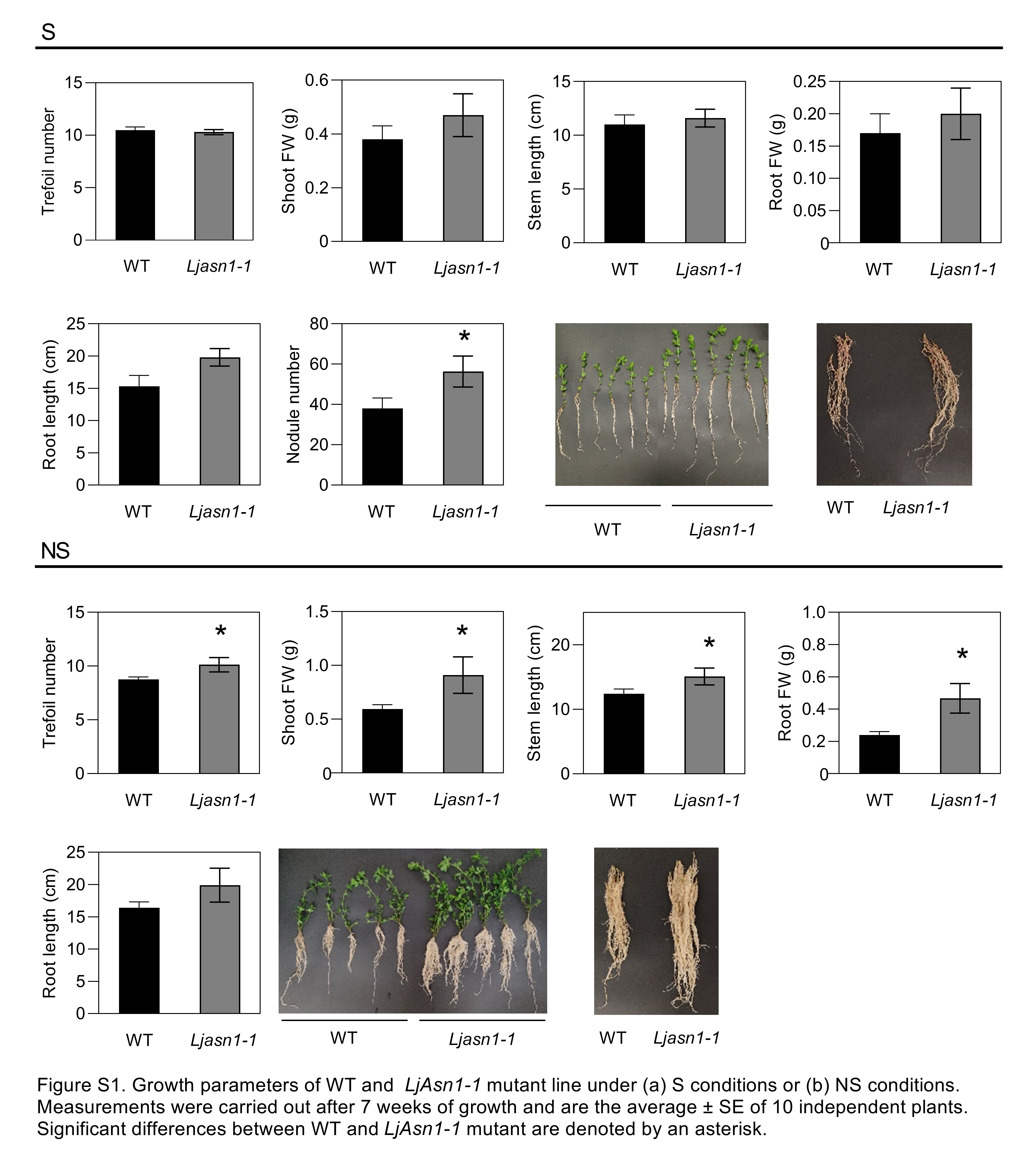

Supplement: Supplementary file 1 — Figure S1: Growth parameters of WT and Ljasn1‐1 mutant line under (a) S conditions or (b) NS conditions. [file PBI-24-4471-s005.tif]

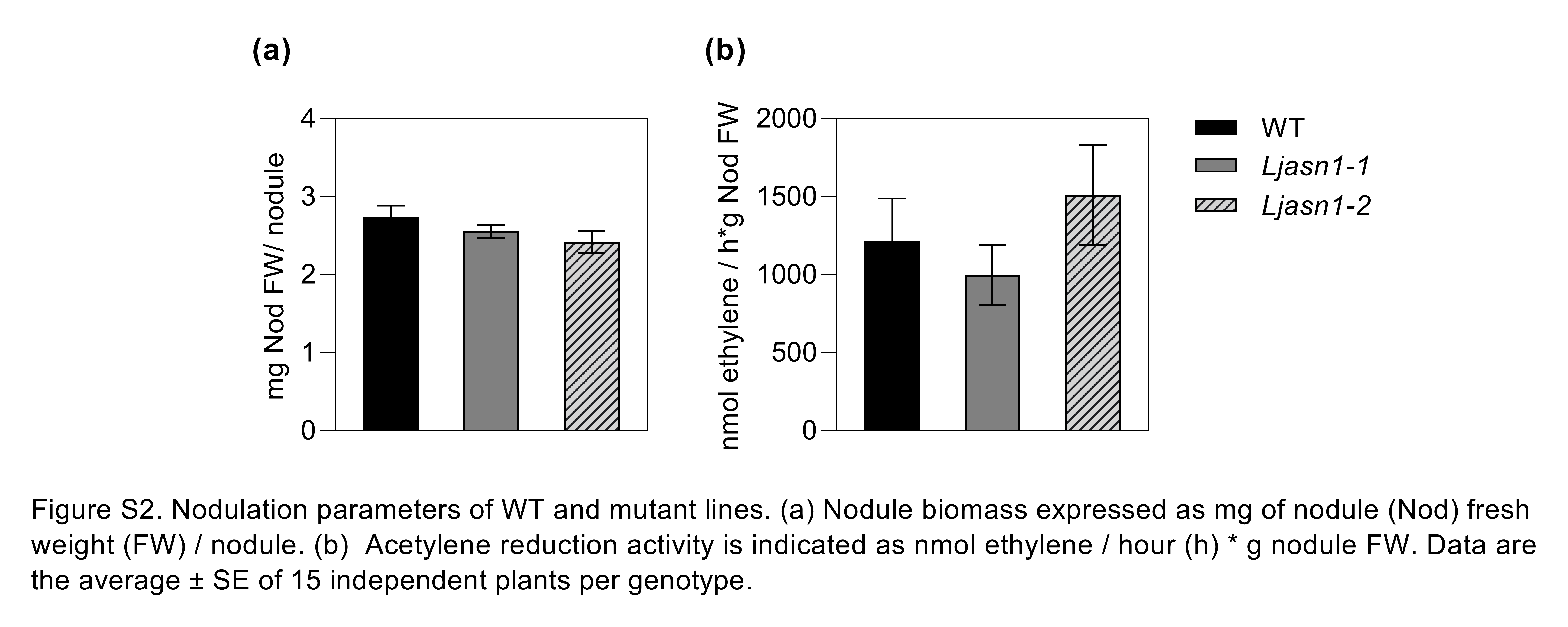

Supplement: Supplementary file 2 — Figure S2: Nodulation parameters of WT and mutant lines. [file PBI-24-4471-s011.tif]

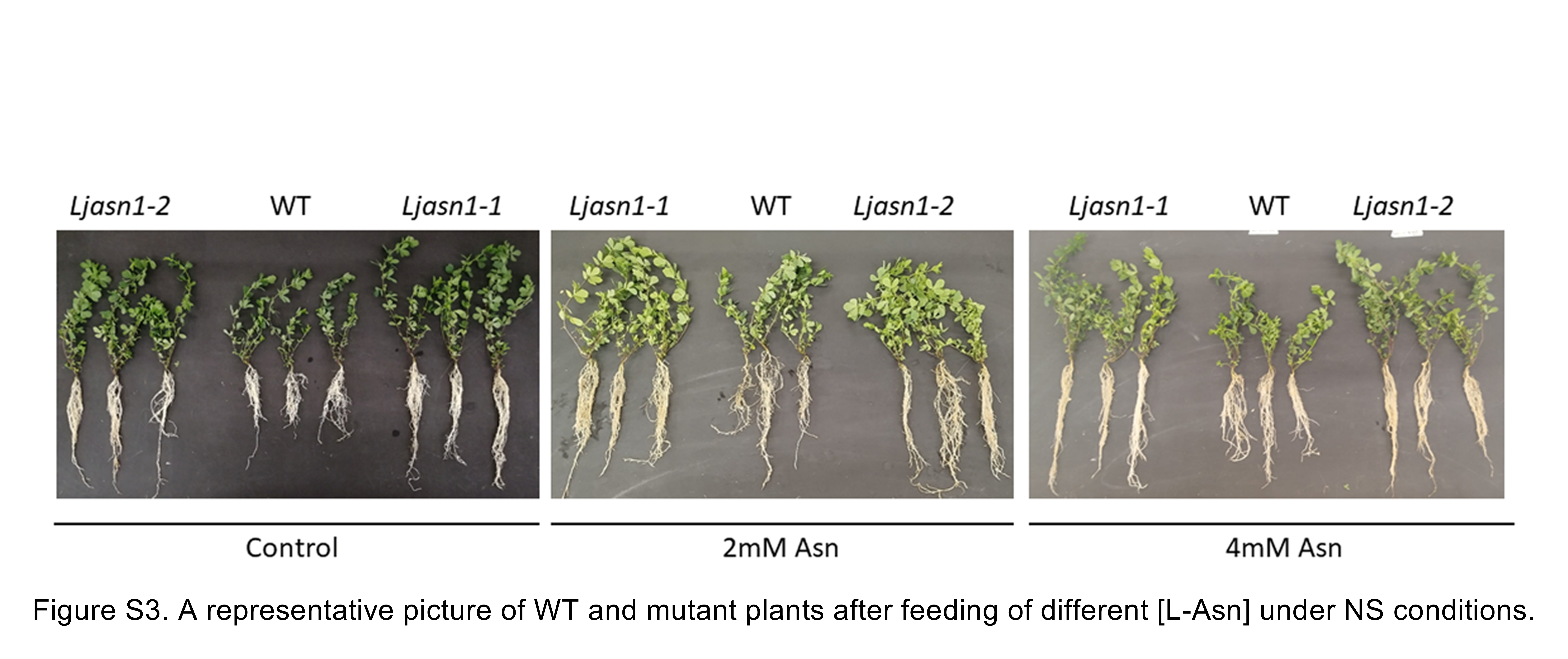

Supplement: Supplementary file 3 — Figure S3: A representative picture of WT and mutant plants after feeding of different [L‐Asn] under NS conditions. [file PBI-24-4471-s003.tif]
