# Supplementary material for: Disruption of Asparagine Synthetase Is Associated to Increased Biomass in Lotus japonicus
Source: Plant Biotechnol J. 2026 Mar 24;24(7):4471–83. doi: 10.1111/pbi.70637 (PMC13278535; doi:10.1111/pbi.70637)
Supplement: Supplementary file 4 — Figure S4: List of enriched GO terms among the shoot and root DEGs under S conditions. [file PBI-24-4471-s010.pdf]

Figure S4. List of genes differentially expressed between *LjAsn1-2* and WT under S conditions. Data are expressed as the log2 fold-change in expression levels between mutant and WT plants. Genes highlighted in yellow have been singularly mentioned in the text of the paper.

	Ontology	GO term	Description	FDR
Shoots	P	GO:0010410	hemicellulose metabolic process	0.0031
	P	GO:0010383	cell wall polysaccharide metabolic process	0.004
	P	GO:0045491	xylan metabolic process	0.0067
	P	GO:0005976	polysaccharide metabolic process	0.0067
	P	GO:0044710	single-organism metabolic process	0.0076
	P	GO:0044036	cell wall macromolecule metabolic process	0.0088
	P	GO:0008643	carbohydrate transport	0.0088
	P	GO:0042546	cell wall biogenesis	0.0088
	P	GO:0009057	macromolecule catabolic process	0.0093
	P	GO:0071554	cell wall organization or biogenesis	0.019
	P	GO:0019748	secondary metabolic process	0.019
	P	GO:0044699	single-organism process	0.02
	P	GO:0009056	catabolic process	0.022
	P	GO:0009620	response to fungus	0.032
	P	GO:0044262	cellular carbohydrate metabolic process	0.044
	C	GO:0031224	intrinsic component of membrane	0.0003
	C	GO:0016021	integral component of membrane	0.0003
	C	GO:0005576	extracellular region	0.0003
	C	GO:0016020	membrane	0.0009
	C	GO:0044425	membrane part	0.0026
	C	GO:0044464	cell part	0.015
	C	GO:0005623	cell	0.015
	C	GO:0031226	intrinsic component of plasma membrane	0.022
	C	GO:0044421	extracellular region part	0.025
Roots	P	GO:0010200	response to chitin	7E-05
	P	GO:0010243	response to organonitrogen compound	0.0001
	P	GO:1901698	response to nitrogen compound	0.0012
	P	GO:0097659	nucleic acid-templated transcription	0.015
	P	GO:0006351	transcription, DNA-templated	0.015
	P	GO:0032774	RNA biosynthetic process	0.015
	P	GO:0016567	protein ubiquitination	0.048
	P	GO:0032446	protein modification by small protein conjugation	0.049
