# Supplementary material for: Disruption of Asparagine Synthetase Is Associated to Increased Biomass in Lotus japonicus
Source: Plant Biotechnol J. 2026 Mar 24;24(7):4471–83. doi: 10.1111/pbi.70637 (PMC13278535; doi:10.1111/pbi.70637)
Supplement: Supplementary file 5 — Figure S5: List of enriched GO terms among the shoot and root DEGs under NS conditions. [file PBI-24-4471-s007.pdf]

Figure S5. List of genes differentially expressed between *LjAsn1-2* and WT under NS conditions. Data are expressed as the log2 fold-change in expression levels between mutant and WT plants. Genes highlighted in yellow have been singularly mentioned in the text of the paper.

	Ontology	GO term	Description	FDR
Shoots	P	GO:0098869	cellular oxidant detoxification	0.000011
	P	GO:1990748	cellular detoxification	0.000011
	P	GO:0090487	secondary metabolite catabolic process	0.000011
	P	GO:0006749	glutathione metabolic process	0.000011
	P	GO:0009407	toxin catabolic process	0.000011
	P	GO:0098754	detoxification	0.000058
	P	GO:0044699	single-organism process	0.00023
	P	GO:0044710	single-organism metabolic process	0.0004
	P	GO:0009636	response to toxic substance	0.00049
	P	GO:0009404	toxin metabolic process	0.00081
	P	GO:0006575	cellular modified amino acid metabolic process	0.0017
	P	GO:0019748	secondary metabolic process	0.0017
	P	GO:0072593	reactive oxygen species metabolic process	0.0049
	P	GO:0048878	chemical homeostasis	0.015
	P	GO:0009812	flavonoid metabolic process	0.021
	P	GO:0051179	localization	0.021
	P	GO:0050896	response to stimulus	0.022
	P	GO:0009056	catabolic process	0.026
	P	GO:0006810	transport	0.029
	P	GO:0051234	establishment of localization	0.03
	P	GO:0042592	homeostatic process	0.032
	P	GO:0006790	sulfur compound metabolic process	0.036
	F	GO:0004497	monooxygenase activity	0.00011
	F	GO:0016491	oxidoreductase activity	0.00015
	**F**	GO:0016765	transferase activity, transferring alkyl or aryl groups	0.00057
	F	GO:0001067	regulatory region nucleic acid binding	0.0017
	F	GO:0000975	regulatory region DNA binding	0.0017
	F	GO:0044212	transcription regulatory region DNA binding	0.0017
	F	GO:0005215	transporter activity	0.0019
	F	GO:0003824	catalytic activity	0.0019
	F	GO:0022857	transmembrane transporter activity	0.011
	F	GO:0046527	glucosyltransferase activity	0.013
	C	GO:0005623	cell	0.000000046
	C	GO:0016020	membrane	0.00000011
	C	GO:0044464	cell part	0.00000011
	C	GO:0005887	integral component of plasma membrane	0.0001
	C	GO:0031224	intrinsic component of membrane	0.00011
	C	GO:0016021	integral component of membrane	0.00011
	C	GO:0044424	intracellular part	0.00011
	C	GO:0005739	mitochondrion	0.00011
	C	GO:0005622	intracellular	0.00017
	C	GO:0005737	cytoplasm	0.00017
	C	GO:0044425	membrane part	0.00047
	C	GO:0005576	extracellular region	0.00052
	C	GO:0043227	membrane-bounded organelle	0.0025
	C	GO:0043231	intracellular membrane-bounded organelle	0.0025
	C	GO:0031226	intrinsic component of plasma membrane	0.0047
	C	GO:0071944	cell periphery	0.0047
	C	GO:0043229	intracellular organelle	0.013
	C	GO:0043226	organelle	0.013
	C	GO:0005886	plasma membrane	0.017
	C	GO:0031090	organelle membrane	0.034
	C	GO:0044459	plasma membrane part	0.037
	C	GO:0019866	organelle inner membrane	0.038

Figure S5. Continued.

Roots	P	GO:0044710	single-organism metabolic process	0.011
	P	GO:0055114	oxidation-reduction process	0.026
	P	GO:0044699	single-organism process	0.026
	P	GO:0002229	defense response to oomycetes	0.026
	P	GO:0002239	response to oomycetes	0.026
	P	GO:0009056	catabolic process	0.026
	F	GO:0016491	oxidoreductase activity	0.01
	C	GO:0005739	mitochondrion	0.00018
	C	GO:0044464	cell part	0.00036
	C	GO:0005623	cell	0.00036
	C	GO:0005737	cytoplasm	0.00053
	C	GO:0044424	intracellular part	0.00053
	C	GO:0044425	membrane part	0.0006
	C	GO:0005622	intracellular	0.00076
	C	GO:0031224	intrinsic component of membrane	0.0012
	C	GO:0016021	integral component of membrane	0.0025
	C	GO:0005887	integral component of plasma membrane	0.0034
	C	GO:0043231	intracellular membrane-bounded organelle	0.0042
	C	GO:0043227	membrane-bounded organelle	0.0042
	C	GO:0043229	intracellular organelle	0.0083
	C	GO:0031226	intrinsic component of plasma membrane	0.0083
	C	GO:0043226	organelle	0.0083
	C	GO:0019866	organelle inner membrane	0.0093
	C	GO:0005743	mitochondrial inner membrane	0.012
	C	GO:0016020	membrane	0.014
	C	GO:0031090	organelle membrane	0.015
	C	GO:0005576	extracellular region	0.032
	C	GO:0031966	mitochondrial membrane	0.036
	C	GO:0044459	plasma membrane part	0.037
	C	GO:0044444	cytoplasmic part	0.046
	C	GO:0005740	mitochondrial envelope	0.046
	C	GO:0071944	cell periphery	0.046
	C	GO:0044455	mitochondrial membrane part	0.049
